# Control of Bone Mass and Remodeling by PTH Receptor Signaling in Osteocytes

**DOI:** 10.1371/journal.pone.0002942

**Published:** 2008-08-13

**Authors:** Charles A. O'Brien, Lilian I. Plotkin, Carlo Galli, Joseph J. Goellner, Arancha R. Gortazar, Matthew R. Allen, Alexander G. Robling, Mary Bouxsein, Ernestina Schipani, Charles H. Turner, Robert L. Jilka, Robert S. Weinstein, Stavros C. Manolagas, Teresita Bellido

**Affiliations:** 1 Division of Endocrinology, Center for Osteoporosis and Metabolic Bone Diseases, Central Arkansas Veterans Healthcare System, University of Arkansas for Medical Sciences, Little Rock, Arkansas, United States of America; 2 Department of Anatomy and Cell Biology, Indiana University School of Medicine, Indianapolis, Indiana, United States of America; 3 Department of Orthopedic Surgery, Harvard Medical School, Orthopedic Biomechanics Laboratory, Beth Israel Deaconess Medical Center, Boston, Massachusetts, United States of America; 4 Endocrine Unit, Massachusetts General Hospital and Harvard Medical School, Boston, Massachusetts, United States of America; 5 Department of Orthopaedic Surgery, Indiana University School of Medicine, Indianapolis, Indiana, United States of America; University of Hong Kong, China

## Abstract

Osteocytes, former osteoblasts buried within bone, are thought to orchestrate skeletal adaptation to mechanical stimuli. However, it remains unknown whether hormones control skeletal homeostasis through actions on osteocytes. Parathyroid hormone (PTH) stimulates bone remodeling and may cause bone loss or bone gain depending on the balance between bone resorption and formation. Herein, we demonstrate that transgenic mice expressing a constitutively active PTH receptor exclusively in osteocytes exhibit increased bone mass and bone remodeling, as well as reduced expression of the osteocyte-derived Wnt antagonist sclerostin, increased Wnt signaling, increased osteoclast and osteoblast number, and decreased osteoblast apoptosis. Deletion of the Wnt co-receptor LDL related receptor 5 (LRP5) attenuates the high bone mass phenotype but not the increase in bone remodeling induced by the transgene. These findings demonstrate that PTH receptor signaling in osteocytes increases bone mass and the rate of bone remodeling through LRP5-dependent and -independent mechanisms, respectively.

## Introduction

Bone remodeling maintains the integrity of the skeleton by continuously replacing packets of old bone with new through the coordinated action of two cell types: osteoclasts that resorb bone, followed by osteoblasts that form bone. The rate of bone remodeling is determined by the number of osteoclasts and osteoblasts; whereas the amount of bone present in the skeleton is determined by the balance between resorption and formation [1]. Osteocytes, former osteoblasts buried within the bone matrix during the process of bone deposition, are distributed throughout bone in numbers that far exceed either osteoclasts or osteoblasts [2], [3]. Osteocytes are connected with each other and with cells on the bone surface via cytoplasmic processes that travel along tunnels within the mineralized bone [4]. Through this extensive network, osteocytes sense variations in the level of mechanical forces acting on bone and respond by signaling to osteoblasts, osteoclasts, or both [5]. Osteocytes also perceive changes in the levels of systemic factors as evidenced by the increased prevalence of osteocyte apoptosis that occurs with glucocorticoid excess or estrogen withdrawal [6]–[11], and increased osteocyte apoptosis may itself decrease bone strength [12]. However, whether hormones influence bone mass or the rate of bone remodeling via actions on osteocytes has heretofore been unknown.

Sclerostin, the product of the Sost gene, is expressed exclusively by osteocytes in bone [13]–[16]. Loss of sclerostin expression in humans results in the high bone mass disorders Van Buchem's disease [17] and sclerosteosis [18], providing compelling evidence that osteocytes can control bone mass. Moreover, targeted deletion of the Sost gene in mice results in increased bone formation and strength [19]; and administration of an anti-sclerostin antibody increases bone formation and restores the bone lost after ovariectomy in rodents [20]. Conversely, transgenic mice overexpressing Sost exhibit low bone mass [13], [21]. Sclerostin acts in a paracrine manner to inhibit bone formation by binding to the Wnt co-receptors LDL receptor-related protein (LRP) 5 and LRP6, thereby antagonizing Wnt actions {13720, 14062, 15337}, such as induction of osteoblastogenesis, stimulation of pre-osteoblast replication, and inhibition of osteoblast apoptosis [25]–[27]. Thus, osteocytes exert negative feedback control of osteoblast number and bone formation via production of sclerostin.

Abundant evidence from humans and experimental animals indicates that parathyroid hormone (PTH) increases the rate of bone resorption, and thereby the rate of bone remodeling. Thus, chronic elevation of PTH levels increases bone resorption [28], [29] whereas patients with hypoparathyroidism [30] or rodents lacking PTH [31], [32] exhibit reduced bone resorption. The currently held view is that PTH stimulates osteoclast formation by binding to its receptor, PTH receptor 1 (PTHR1), on stromal/osteoblastic cells and thereby increases production of receptor activator of NFkB ligand (RANKL) and macrophage colony stimulating factor (M-CSF) and suppresses the RANKL decoy receptor osteoprotegerin (OPG) [33]–[36]. Consistent with this notion, removal of the PTH responsive region of the RANKL gene is sufficient to reduce the rate of bone resorption, mimicking the effects of hypoparathyroidism [37]. Chronic PTH elevation also increases osteoblast number and bone formation. This may occur indirectly through stimulation of bone resorption which releases growth factors embedded in the bone matrix and these in turn promote osteoblast formation [38]. Intermittent administration of PTH as a therapy to induce bone anabolism also increases osteoblast number, but the mechanisms are thought to be different from those involved with chronic PTH elevation [29], [39].

Chronic PTH elevation, as in hyperparathyroidism, causes loss of cortical bone. Cancellous bone is also lost with secondary hyperparathyroidism caused by dietary calcium deficiency, but it is preserved or even increased in primary hyperparathyroidism or with activating mutations of PTHR1 [40], [41]. Thus, PTH always increases the rate of bone remodeling, but it can result in either loss or gain of cancellous bone mass depending on the balance between resorption and formation. The mechanisms underlying these different outcomes of PTH action are unknown.

PTH related peptide (PTHrP) binds to PTHR1 and has important actions during development [42]. Recent genetic studies in mice demonstrate that PTHrP also acts postnatally to control bone mass [43], [44]. Thus, mice with PTHrP haploinsufficiency, or with deletion of the PTHrP gene specifically from osteoblasts, exhibit reduced bone formation due to increased osteoblast apoptosis. In addition, the number of osteoclasts is reduced in these animals most likely because of reduced RANKL expression. Thus, activation of PTHR1 by either PTH or PTHrP increases the rate of bone remodeling [45].

We and others have recently determined that PTH inhibits the expression of the osteocyte specific gene Sost, raising the possibility that the hormone stimulates bone formation via direct actions on osteocytes [15], [46]. Consistent with this idea, expression of PTHR1 has been demonstrated in osteocytes [47]. Here we demonstrate that activation of PTHR1 signaling exclusively in osteocytes in transgenic mice is sufficient to decrease sclerostin expression, increase Wnt signaling, and increase bone mass. Unexpectedly, PTHR1 activation in osteocytes also accelerates the rate of bone remodeling. Remarkably, deletion of the Wnt co-receptor LRP5 attenuates the high bone mass phenotype induced by the transgene, but does not affect the increased remodeling. Thus, PTH signaling in osteocytes stimulates the accrual of bone mass and increases the rate of bone remodeling by LRP5-dependent and -independent mechanisms, respectively. Hence, the skeletal effects of PTH and PTHrP may be due in part to previously unrecognized actions of PTHR1 activation in osteocytes.

## Results

### Mice Expressing a Constitutively Active PTHR1 in Osteocytes Exhibit High Bone Mass

**Figure 1:**
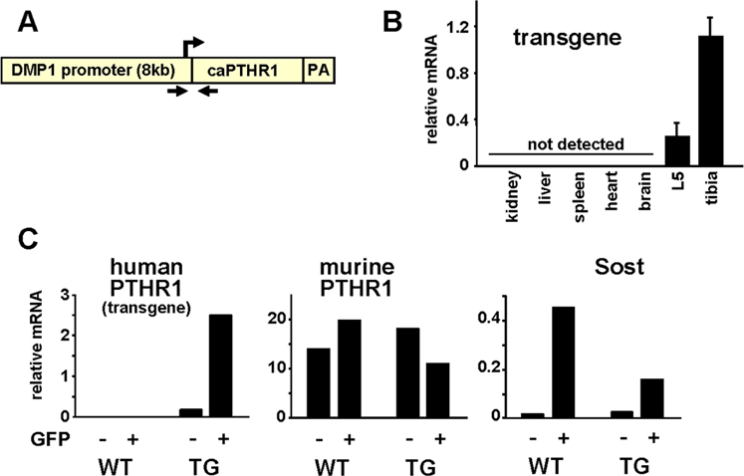
Generation of DMP1-caPTHR1 Transgenic Mice. (A) Schematic representation of the DMP1-caPTHR1 transgene. The transgene contains the cDNA encoding the H223R constitutively active mutant of PTHR1 (caPTHR1) inserted downstream from a 12-kb DNA fragment containing 8 kb of the murine DMP1 5′-flanking region, first exon, first intron, and 17 bp of exon 2; a synthetic polyadenylation site was inserted downstream from the caPTHR1 sequence. Arrows indicate the sites recognized by primers used for genotyping. (B) Quantitative RT-PCR analysis of DMP1-caPTHR1 mRNA from the 5^th^ lumbar vertebrae (L5), tibia, and soft tissues of 10.5-week-old mice, normalized to the housekeeping gene ribosomal protein S2. Bars represent the mean±SD of 4 mice. (C) Expression of the human PTHR1, murine PTHR1, and Sost determined by quantitative RT-PCR in freshly isolated osteoblast-enriched (GFP−) and osteocyte-enriched (GFP+) cell preparations obtained from neonatal mice without (WT) or with (TG) the DMP1-caPTHR1 transgene.

To determine whether hormonal signaling in osteocytes is sufficient to alter skeletal homeostasis, we generated transgenic mice expressing a constitutively active form of human PTHR1 [48] specifically in osteocytes using the dentin matrix protein-1 (DMP1) promoter (DMP1-caPTHR1 mice) (**Figure 1A**). This promoter was previously shown to direct osteocyte-specific expression of transgenes in mice [5], [49]–[52]. Expression of the DMP1-caPTHR1 transgene was detected in bone tissues (L5 vertebra and tibia), but not in kidney, liver, spleen, heart, or brain (**Figure 1B**). To confirm osteocyte-specific expression of the transgene, hemizygous DMP1-caPTHR1 transgenic mice were crossed with homozygous DMP1-GFP transgenic mice in which GFP is expressed only in osteocytes [15], [49], [50], and gene expression was examined in GFP-positive and GFP-negative cell populations obtained from neonatal calvaria as detailed in **Materials and Methods** (**Figure 1C**). In two independent experiments (**Figures 1C** **and S1A**), transcripts for the DMP1-caPTHR1 transgene were found only in cells obtained from DMP1-caPTHR1 transgenic mice and were highly enriched in the GFP-positive, osteocyte fraction. In contrast, murine PTHR1 mRNA levels were similar in GFP-positive and GFP-negative cell populations in mice with and without the DMP1-caPTHR1 transgene. Expression of the osteocyte-specific gene Sost was enriched in the GFP-positive cells obtained from mice with or without the DMP1-caPTHR1 transgene. Furthermore, consistent with previous findings demonstrating that PTH inhibits Sost expression [15], [46], Sost mRNA was lower in osteocytes obtained from DMP1-caPTHR1 mice. These results demonstrate that the DMP1-caPTHR1 transgene was expressed exclusively in osteocytes.

**Figure 2:**
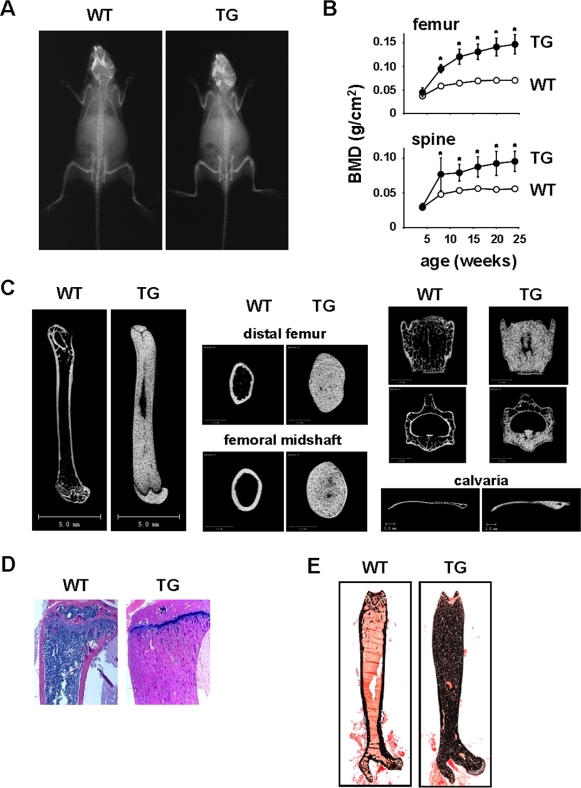
DMP1-caPTHR1 Mice Exhibit High Bone Mass. (A) Radiographs of 4-week-old female wild type (WT) and DMP1-caPTHR1 transgenic (TG) mice. (B) Femoral and spinal BMD in WT and TG female mice, measured at 4 wk intervals up to 24 wks of age. Symbols represent the mean±SD of 5 mice. * p<0.05 vs. WT mice at each time point. (C) Representative longitudinal and cross-sectional micro-CT images of femur, 4th lumbar vertebra, and calvaria obtained from 10.5-week-old female WT and TG mice. (D) Hematoxylin and eosin staining of tibial and (E) von Kossa staining of femoral bone sections from 10.5-week-old WT and TG mice.

DMP1-caPTHR1 transgenic mice were born at the expected Mendelian frequency, were fertile, exhibited normal size and weight (**Figure S1B**), but displayed increased radiodensity in regions of the skull and long bones at 4 weeks of age (**Figure 2A**). Serial measurement of bone mineral density (BMD) by dual-energy x-ray absorptiometry (DEXA) revealed a remarkable and progressive increase in bone mass in the appendicular and axial skeleton in transgenic mice of both sexes compared to wild type littermates (**Figure 2B** **and S1C**). Femoral BMD was 63% higher than wild type littermates at 8 weeks of age and 109% higher at 24 weeks. Increased bone mass in the transgenic mice was maintained until at least 8 months of age (**Figure S1D**). Micro-CT of femur and vertebra of 10-week-old transgenic mice confirmed the high bone mass phenotype (**Figure 2C**). Cross-sectional area of the femur at both the distal metaphysis and midshaft was elevated by approximately 2-fold. Increased bone was also observed in the calvaria, a bone that is formed by intramembranous ossification (**Figure 2C**). Abundant bone tissue and increased mineral was revealed by staining with hematoxylin & eosin or von Kossa (**Figures 2D and 2E**). This high bone mass phenotype was also present in a second line of DMP1-caPTHR1 transgenic mice (**Figure S1E**).

### DMP1-caPTHR1 Mice Exhibit Elevated Bone Remodeling

**Figure 3:**
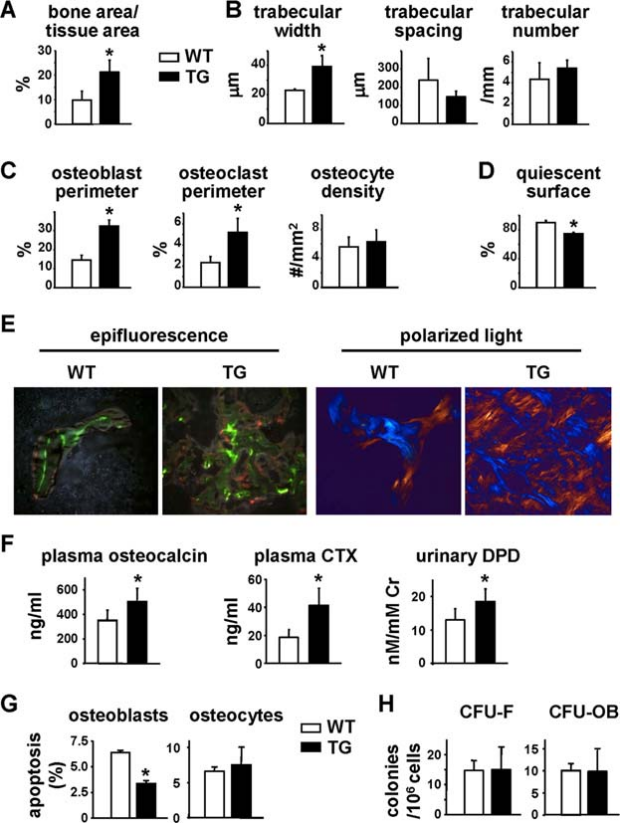
DMP1-caPTHR1 Transgenic Mice Have Increased Bone Turnover. (A–D) Histomorphometric measurements were determined in the distal femur, excluding the growth plate, of 3.5-week-old DMP1-caPTHR1 mice (TG) and wild type littermates (WT). Both sexes were included. Bone area (A), trabecular architecture (B), osteoblast perimeter, osteoclast perimeter, and osteocyte density (C), and quiescent surface (D) are shown. (E) Distal femoral cancellous bone of WT or TG mice viewed with epifluorescence to reveal calcein and alizarin labels or with polarized light with a rose quartz plate to reveal collagen architecture (×200). (F) Serum osteocalcin, serum CTX, and urinary DPD were measured in 3.5- to 5-week-old mice. (G) Osteoblast and osteocyte apoptosis measured in femoral sections from 3.5-week-old mice. (H) CFU-F and CFU-OB obtained from bone marrow cells of 4.5-week-old mice. Bars represent the mean±SD of 3–5 mice. * p<0.05 vs. WT mice.

The high bone mass phenotype of DMP1-caPTHR1 transgenic mice was present as early as 3 days after birth, had progressed by 3.5 weeks of age (**Figure S2**), and appeared maximal before 12 weeks of age (**Figure S3**). To determine the cellular basis of the increased bone mass, we performed histomorphometric measurements of cancellous bone of the distal femur in 3.5-week-old mice. This age was selected for histomorphometric analysis because, in contrast to mice at older ages, a large amount of cancellous bone surface was available for analysis and the cellular processes leading to increased bone mass must have still been occurring. Cancellous bone area and trabecular width were increased in femoral bone of the transgenic mice (**Figure 3A and B** **, and Table S1**); however, trabecular spacing and number were not significantly different from wild type littermates. Both osteoblast and osteoclast perimeters were elevated by more than 2-fold in transgenic mice, whereas the number of osteocytes per cancellous bone area (osteocyte density) was unchanged (**Figure 3C**). This increase in the numbers of osteoclasts and osteoblasts, and thus the rate of bone remodeling, was reflected by a decrease in quiescent surface (**Figure 3D**). The DMP1-PTHR1 transgenic mice also exhibited diffuse incorporation of the fluorochromes (**Figure 3E**). Therefore, quantification of the rate of bone formation was not possible. The presence of diffuse labels could be due to rapid bone formation leading to woven bone, which was indeed revealed by polarized light microscopy (**Figure 3E**). However, abnormal mineralization cannot be excluded since osteoid surface was also elevated in the transgenic mice (**Table S1**). Nevertheless, and consistent with the elevated osteoblast number and expected high bone formation rate, circulating osteocalcin was elevated in DMP1-caPTHR1 mice (**Figure 3F**). Moreover, in line with the increase in osteoclast perimeter, plasma levels of carboxy-terminal crosslinked telopeptide of type I collagen (CTX) and urinary levels of deoxypyridinoline (DPD), markers of bone resorption, were also elevated in the transgenic mice (**Figure 3F** **)**. No significant changes were found in circulating levels of calcium, inorganic phosphate, or PTH (**Figure S4**), measured as indicated in the **Supplementary Methods** (Text S1) section. The increase in osteoblast number induced by the transgene was associated with a decrease in the prevalence of osteoblast apoptosis (**Figure 3G**). In contrast, osteocyte survival was not affected (**Figure 3G**). The number of mesenchymal progenitors or committed osteoblast progenitors in the bone marrow, as measured by colony forming units (CFU)-fibroblasts (CFU-F) or CFU-osteoblasts (CFU-OB), respectively, was not increased in the transgenic mice (**Figure 3H**). These results indicate that PTHR1 activation in osteocytes leads to an increased rate of bone remodeling with a positive balance resulting in more bone, associated with reduced prevalence of osteoblast apoptosis.

### Sost and Sclerostin Expression are Reduced and Wnt Signaling is Increased in DMP1-caPTHR1 Mice

**Figure 4:**
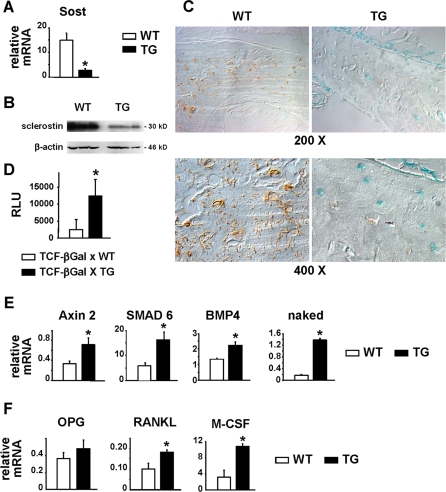
Sost and Sclerostin Expression are Decreased and Wnt Signaling is Increased in DMP1-caPTHR1 Transgenic Mice. (A) Quantitative RT-PCR analysis of Sost mRNA in tibia from 9-week-old DMP1-caPTHR1 mice and wild type littermates (n = 3). Both sexes were included. (B) Sclerostin protein levels were determined by Western blot analysis of lysates from the 6th lumbar vertebrae of 9-week-old wild type (WT) and DMP1-caPTHR1 (TG) mice. Each lane contains protein lysate from a single mouse. (C) Anti-sclerostin immunohistochemistry in ulnae sections from 10.5-week-old WT and TG mice. (D) β-galactosidase activity was measured in lysates of femurs obtained from 4.5-week-old TCF-βgal reporter mice with and without the DMP1-caPTHR1 transgene, and is expressed as relative luminescence units (RLU)/200 mg protein (n = 3–5 mice/group). Quantitative RT-PCR analysis of the indicated Wnt target genes (E), and OPG, RANKL, and M-CSF (F), in tibia from 8-week-old DMP1-caPTHR1 mice and wild type littermates. Bars represent the mean±SD of 3 mice. * p<0.05 vs. WT mice.

Quantitative RT-PCR and *in situ* hybridization demonstrated an increase in the mRNAs encoding osteocalcin, collagen 1a1, and osteopontin in bones from DMP1-caPTHR1 mice compared to wild type littermates (**Figure S5A and B**, and **Supplementary Methods** section). Furthermore, expression of the osteoclast-specific genes cathepsin K, tartrate resistant acid phosphatase (TRAPase), and calcitonin receptor was also elevated in the transgenic animals (**Figure S5C**). On the other hand, DMP1-caPTHR1 transgenic mice exhibited a striking decrease in Sost transcripts and sclerostin protein, as determined by quantitative RT-PCR, Western blot analysis, and immunohistochemistry (**Figure 4A, B and C**). Increased β-galactosidase activity in bones from the DMP1-caPTHR1 mice crossed with Wnt-responsive reporter mice (TCF-βGal) demonstrated that decreased Sost and sclerostin expression were associated with elevated Wnt signaling *in vivo* (**Figure 4D**). Furthermore, expression of the Wnt/β-catenin target genes Axin 2, SMAD6, BMP4, and naked [53]–[55] was elevated in DMP1-caPTHR1 transgenic mice (**Figure 4E**). OPG, another gene regulated by Wnt/β-catenin signaling [56], [57], was also slightly elevated, although this increase did not reach statistical significance (**Figure 4F**). In contrast, RANKL and M-CSF, cytokines that control the magnitude of osteoclast formation, were significantly increased in DMP1-caPTHR1 transgenic mice (**Figure 4F**).

### LRP5 Signaling Contributes to the Increased Bone Mass, but not to the Increased Remodeling, of DMP1-caPTHR1 Mice

**Figure 5:**
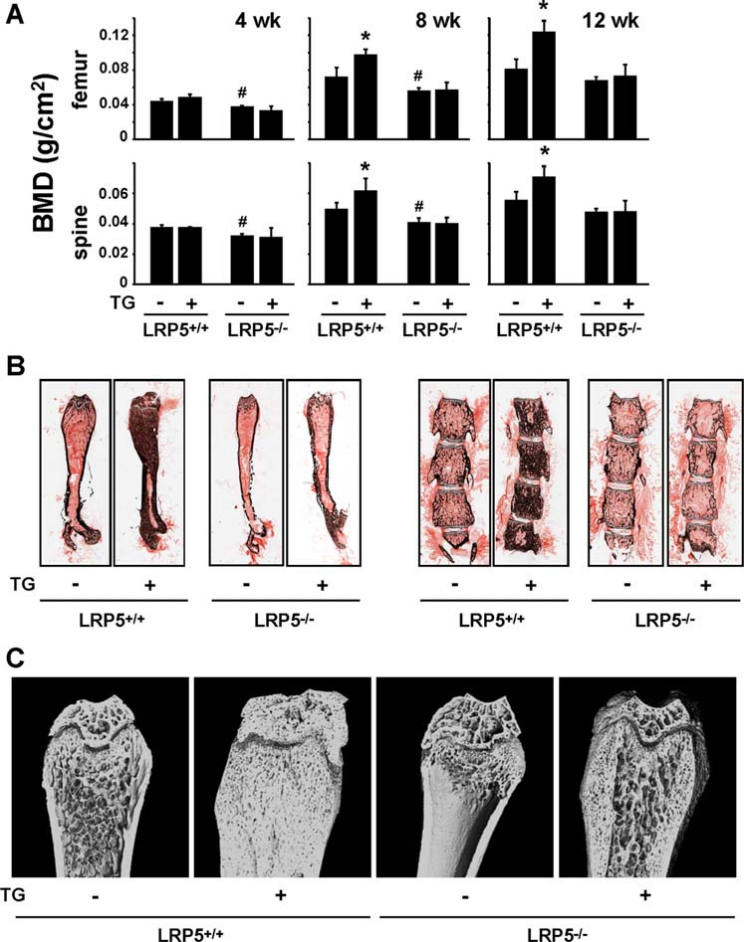
The High Bone Mass Phenotype of the DMP1-caPTHR1 Mice is Ameliorated in Mice Lacking the Wnt Co-Receptor LRP5. (A) Serial analysis of BMD in the femur and spine of LRP5+/+ and LRP−/− mice, with and without the DMP1-caPTHR1 transgene. Both sexes were included. Bars represent the mean±SD of 3–5 mice/group. * p<0.05 vs. LRP5+/+ mice without the transgene. # p<0.05 vs. LRP+/+ mice without the transgene. (B) Von Kossa staining of femurs and lumbar vertebrae of 12-week-old LRP5+/+ and LRP−/− mice, with and without the DMP1-caPTHR1 transgene. (C) High resolution micro-CT scans of distal femora from LRP5+/+ and LRP−/− mice, with and without the DMP1-caPTHR1 transgene.

**Figure 6:**
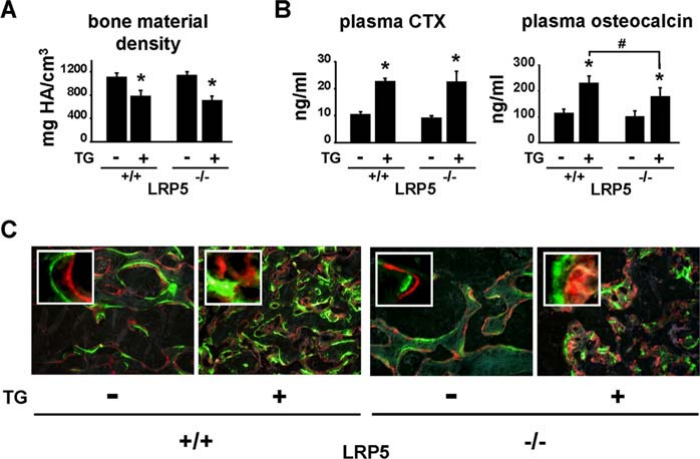
The High Bone Remodeling Phenotype of DMP1-caPTHR1 Mice does not Require LRP5. (A) Osteocalcin and CTX measured in plasma from 12-week-old LRP5+/+ and LRP−/− mice, with and without the DMP1-caPTHR1 transgene. Both sexes were included. (B) Bone material density as determined by micro-CT analysis of femurs from LRP5+/+ and LRP−/− mice, with and without the DMP1-caPTHR1 transgene. Bars represent the mean±SD of 3–5 mice/group. * p<0.05 vs. LRP5+/+ mice without the transgene; # p<0.05 vs. LRP5+/+ mice with the transgene. (C) Representative images of histologic sections showing calcein and alizarin double labeling in femurs from LRP5+/+ and LRP−/− mice with and without the DMP1-caPTHR1 transgene.

To determine whether inhibition of Sost gene expression in osteocytes and the resulting increase in Wnt signaling was indeed responsible for the high bone mass phenotype exhibited by DMP1-caPTHR1 mice, these mice were crossed with mice lacking the Wnt co-receptor LRP5 (LRP5−/−), which display a low bone mass phenotype due to reduced osteoblast number and activity [58]–[60]. Sost mRNA expression in DMP1-caPTHR1;LRP5−/− mice was still suppressed compared to LRP5−/− mice lacking the transgene (0.37±0.23 versus 2.00±1.16, p<0.05), eliminating the possibility that the absence of LRP5 interfered with the ability of the transgene to decrease Sost in osteocytes. Serial BMD measurements confirmed the progressive increase in bone mass between 4 and 12 weeks of age in the femur and spine of DMP1-caPTHR1 mice in a LRP5+/+ background compared to wild type littermates (**Figure 5A**). In contrast, DMP1-caPTHR1 transgenic mice in a LRP5−/− background exhibited BMD similar to LRP5−/− littermates lacking the transgene (**Figure 5A**). The blunting of the effect of the transgene in mice lacking LRP5 was confirmed by Von Kossa staining and high resolution micro-CT at 12 weeks of age (**Figure 5B and C**). Micro-CT images also revealed that the transgene was still able to increase cancellous bone in animals lacking LRP5, however this effect was greatly reduced compared to animals expressing LRP5 (**Figure 5C**). This persistent increase in bone induced by the transgene in animals lacking LRP5 could be due to increased Wnt signaling through LRP6. In addition, bone material density as measured by micro-CT was decreased by the transgene regardless of the LRP5 genotype (**Figure 6A**). This may explain why the increased cancellous bone in DMP1-caPTHR1;LRP5−/− mice detected by micro-CT did not result in increased BMD as measured by DEXA (**Figure 5A**).

Despite the reduced ability of the transgene to increase bone mass in the absence of LRP5, circulating levels of CTX and osteocalcin remained elevated in DMP1-caPTHR1;LRP5−/− mice (**Figure 6B**). However, the increase in osteocalcin was significantly lower than the one induced by the transgene in the LRP5+/+ background. Consistent with a high rate of bone resorption, the DMP1-caPTHR1;LRP5−/− mice still exhibited increased cortical porosity (**Figure 5B and C**). Furthermore, DMP1-caPTHR1;LRP5−/− mice showed the same abundant and diffuse fluorochrome incorporation observed in DMP1-caPTHR1 mice with intact LRP5 signaling (**Figure 6C**). This phenomenon, as well as the reduced bone material density, may be due to the formation of woven bone [61], abnormal mineralization [51], or both.

Taken together, these results suggest that activation of the PTH receptor in osteocytes results in two separate effects: one that leads to increased bone mass and that depends in part on LRP5 signaling to favor bone formation, and another that results in increased bone remodeling independent of LRP5 signaling.

## Discussion

**Figure 7:**
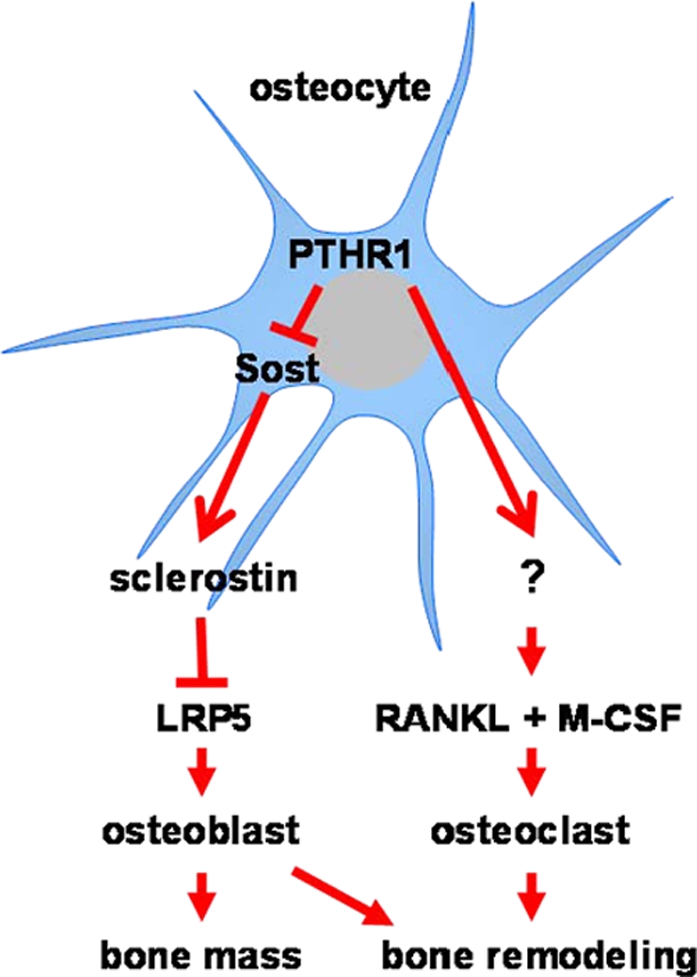
PTHR1 Signaling in Osteocytes Leads to Increased Bone Mass and Remodeling via Distinct Mechanisms. In the proposed model, PTHR1 signaling in osteocytes activates at least two distinct pathways: one leading to increased bone mass and the other leading to increased bone remodeling. Suppression of Sost/sclerostin and activation of LRP5 signaling increase osteoblast numbers and are required for the increase in bone mass. The elevation of bone resorption is independent of the Sclerostin/LRP5 pathway. The question mark indicates uncertainty of the cellular source of RANKL and M-CSF (osteocytes versus stromal/osteoblastic cells).

The concept that PTH can act directly on osteocytes was proposed in the 1970's based on evidence that injected radiolabeled-PTH localizes in osteocytes and that the hormone changes osteocyte morphology [62], [63]. Recently we and others have shown that PTH suppresses the expression of the osteocyte-specific product sclerostin, an antagonist of Wnt signaling [15], [46]. This evidence, together with the well documented role of Wnt signaling in osteoblastogenesis, suggested a mechanism by which PTH may control the production of osteoblasts through actions on osteocytes. In the present report, we have addressed the functional consequences of PTH actions in osteocytes by taking advantage of a fragment of the DMP1 promoter that confers osteocyte-specific expression of transgenes [5], [49], [52], [64]. The evidence presented herein demonstrates that PTH receptor signaling exclusively in osteocytes is sufficient to increase osteoblast number and dramatically increase bone mass via down-regulation of sclerostin and thereby elevation of LRP5 signaling. Remarkably, activation of PTHR1 in osteocytes also caused an increase in bone resorption, and thereby the rate of bone remodeling. This effect of PTH was previously attributed to its actions on stromal/osteoblastic cells [34], [41]. Thus, our findings reveal for the first time that osteocytes could be the targets and mediators of the two most important effects of PTH on bone (**Figure 7**). Whether this is the case outside the context of the genetic manipulation used here will await osteocyte-specific deletion of the PTH receptor.

PTH can clearly increase bone mass when administered intermittently, and this effect has been attributed by us to a direct and potent anti-apoptotic effect of the hormone on osteoblasts [29], [39], [65]. In the present report, we determined that the increased osteoblast number and bone formation caused by PTH signaling exclusively in osteocytes is also associated with decreased osteoblast apoptosis. Attenuation of osteoblast apoptosis in our model can be readily explained by unleashing Wnt signaling secondary to suppression of Sost expression. Indeed, several studies demonstrate that Wnt signaling increases osteoblast number and bone formation at least in part by inhibiting osteoblast apoptosis. Specifically, the increased bone mass observed in mice deficient in the Wnt antagonist secreted frizzled related protein-1 (SFRP-1), or mice expressing a LRP5 mutant (G171V) unable to bind sclerostin, is associated with decreased osteoblast and osteocyte apoptosis [66]–[68]. Moreover, direct activation of Wnt signaling *in vitro* prevents apoptosis of osteoblastic cells [27].

Besides the anti-apoptotic effects of Wnts in the increased bone mass of the DMP1-caPTHR1 mice, the unleashing of Wnt signaling in this model may have had additional effects on cells of the osteoblastic lineage. We did not find an increase in the number of CFU-OB, indicating that such additional effects did not influence the very early stages of osteoblastogenesis. It is entirely feasible, however, that Wnts also increased osteoblast number by attenuating the apoptosis, stimulating the differentiation, or increasing the proliferation of the progeny of CFU-OB, or a combination of these mechanisms.

Targeted activation of the PTH receptor exclusively in osteocytes also increased osteoclast number and bone resorption and these changes were accompanied by an elevation of the levels of RANKL and M-CSF, the two essential signals for osteoclastogenesis. Whether osteocytes are the source of these cytokines, or whether an osteocyte-derived product stimulated the expression of these cytokines in a paracrine fashion by altering their production in stromal/osteoblastic cells, awaits further studies.

The results of the present studies with genetically modified mice could well be relevant to the mechanisms underlying the changes in bone cells that occur with hyperparathyroidism. Several skeletal features of the DMP1-caPTHR1 mice are similar to those found in patients with hyperparathyroidism, including a high rate of bone remodeling, increased cancellous bone, and increased cortical porosity [69]. Indeed, the cortical porosity and cancellous bone were increased to such an extent that the junction between cortical and cancellous bone could not be distinguished (**Figure 2**). Erosion of the cortex, as well as the presence of woven bone, is also seen in patients with secondary hyperparathyroidism in renal osteodystrophy [69]. Therefore, some of the skeletal changes that occur in humans with high levels of PTH may be due to direct actions of the hormone on osteocytes.

The striking bone gain in DMP1-caPTHR1 mice and the blunting of this effect in mice lacking LRP5 suggest that PTH signaling in osteocytes promotes a positive balance within each remodeling unit via suppression of Sost. Consistent with this contention, in preliminary studies we found that DMP1-caPTHR1 mice that also harbor a DMP1-Sost transgene do not exhibit high bone mass, indicating that suppression of Sost is indeed required for the anabolic effect of the DMP1-caPTHR1 transgene (data not shown).

In contrast to our findings demonstrating dramatic bone anabolism with PTHR1 signaling exclusively in osteocytes, chronic elevation of PTH systemically leads to bone loss or to only a modest increase in cancellous bone. This difference may be explained by actions of the hormone on mature osteoblasts and osteoblast precursors that counter-balance osteocyte-mediated effects on bone formation. Specifically, the prevalence of osteoblast apoptosis is decreased in DMP1-caPTHR1 mice but not with chronic elevation of PTH [29]. Yet, sclerostin is suppressed, and Wnt signaling is elevated, in both conditions [15]. Therefore, it appears that Wnt signaling is not able to inhibit apoptosis in a cell in which the PTHR1 is continuously activated. Consistent with this, osteocyte apoptosis was not suppressed in DMP1-caPTHR1 mice likely because the pro-survival effects of Wnts were overridden by the simultaneous PTHR1 signal, whereas osteoblast apoptosis was suppressed because osteoblasts receive only Wnt pro-survival signals. Chronic elevation of PTH may also suppress osteoblast differentiation and lead to accumulation of immature osteoblasts. This effect could account for the marrow fibrosis found in severe hyperparathyroidism [70] and in transgenic mice expressing a constitutively active PTHR1 under the control of a collagen 1a1 promoter fragment [41], [71]. Maturational arrest of osteoblastic cells was not observed in DMP1-caPTHR1 mice, indicating that this effect is due to PTHR1 activation in cells other than osteocytes.

Resorption was still elevated in DMP1-caPTHR1 mice in a LRP5-null background, supporting the contention that increased Wnt signaling in these mice does not contribute to the increased rate of bone remodeling. This is consistent with evidence that models of elevated Wnt signaling, such as SFRP-1 knockout mice or G171V-LRP5 transgenic mice, do not exhibit increased bone resorption [66], [67]. Moreover, administration of an anti-sclerostin antibody as well as deletion of the Sost gene, maneuvers which increase Wnt signaling through both LRP5 and LRP6, increased bone formation but did not alter bone resorption [19], [20].

In contrast to chronic elevation of PTH, daily injections of the hormone induce bone anabolism. This mode of administration of PTH is associated with a transient suppression of sclerostin [15], [46], which could result in increased Wnt signaling. Although daily injections of PTH induced equivalent increases in femoral BMD in LRP5-null and wild type mice [72], [73], these animals retained LRP6. Thus, the possibility that suppression of sclerostin contributes to the anabolic action of intermittent PTH administration will require additional studies.

The decreased bone formation exhibited by mice lacking PTHrP in osteoblastic cells demonstrates that under physiological conditions osteoblast/osteocyte-derived PTHrP promotes bone formation in part by stimulating osteoblast differentiation and survival [44]. Our studies raise the possibility that PTHrP promotes bone formation postnatally via suppression of sclerostin and stimulation of Wnt signaling. In particular, PTHrP might contribute to the maintenance of skeletal mass by mechanical forces. Indeed, PTHrP expression is increased in response to mechanical stimulation of osteoblastic and osteocytic cell lines [74]. And, we have shown that the anabolic response of bone loading is associated with reduced sclerostin expression and that it requires LRP5 signaling [72], [73]. Taken together these findings raise the possibility that local increase in PTHrP induced by mechanical loading activates PTHR1 in osteocytes, thereby reducing sclerostin expression and increasing bone formation.

In conclusion, this study expands the prevailing view of osteocytes as mediators of the response of bone to mechanical forces to one in which these cells also orchestrate changes in bone mass and the rate of remodeling in response to hormones.

## Materials and Methods

### Generation of DMP1-caPTHR1 Transgenic Mice and Cross with LRP5-Null Mice

DMP1-caPTHR1 transgenic mice were generated using a DNA construct encoding the H223R mutant of the human PTHR1 [48] inserted downstream from a 12 kb DNA fragment containing 8 kb of the 5′-flanking region, the first exon, the first intron, and 17 bp of exon 2 of the murine DMP1 gene [49]. In addition, a 140 bp fragment containing the rabbit beta-globin polyadenylation sequence was inserted downstream from the H223R cDNA. The DNA sequence of the transgene is available upon request. Transgenic mice were produced by microinjection of purified DNA into pronuclei of C57BL/6 mice at the transgenic mouse core facility of the University of Arkansas for Medical Sciences. DMP1-caPTHR1 transgenic mouse colonies were maintained by breeding mice hemizygous for the transgene with wild type C57BL/6 mice. All DMP1-caPTHR1 mice used in these studies were hemizygous for the transgene. LRP5^−/−^ mice [58] were crossed with DMP1-caPTHR1 mice and the progeny were intercrossed to obtain LRP5^+/+^ and LRP5^−/−^ mice with and without the DMP1-caPTHR1 transgene. All mice were fed a regular diet (Harlan/Teklad #7001) and water ad libitum and maintained on a 12-hr light/dark cycle. Protocols involving genetically modified mice and their wild type littermates were approved by the Institutional Animal Care and Use Committees of the University of Arkansas for Medical Sciences and the Central Arkansas Veterans Healthcare System.

### Analysis of Skeletal Phenotypes

Radiographic images were obtained from anesthetized mice using a Faxitron X-ray system (AxR model MIIONH, Faxitron X-ray Corp., Wheeling, IL) [75]. BMD was determined by DEXA using a PIXImus densitometer (G.E. Medical Systems, Lunar Division, Madison, WI) as previously described [28]. BMD measurements included the entire thoracic and lumbar spine (spinal BMD) or the entire femur (femoral BMD). For micro-CT analysis, bones were dissected, cleaned of soft tissue and stored in 70% ethanol until analyzed using a Scanco model µCT40 instrument (Scanco Medical AG, Basserdorf, Switzerland) [76]. For histomorphometric analysis, femurs were dissected, fixed, and embedded in methyl methacrylate. Static histomorphometric analysis of non-decalcified bone sections was performed in the distal femur, avoiding the growth plate, as previously described [6]. Fluorochrome labeling of the bones was performed by intraperitoneal injections of calcein (20 mg/kg, Sigma Chemical, St. Louis, MO, USA) and alizarin (20 mg/kg, Sigma) administered 8 and 3 days before sacrifice, respectively, as previously described [72]. The terminology and units used are those recommended by the Histomorphometry Nomenclature Committee of the American Society for Bone and Mineral Research [77]. Detection of apoptotic osteoblasts and osteocytes by *in situ* nick-end labeling was performed as previously described [6], [78]. Mineralized bone was visualized in non-decalcified, plastic-embedded sections using the von Kossa stain, and decalcified, paraffin embedded bone sections were stained with hematoxylin and eosin, using standard histological procedures.

### Bone Turnover Markers

Plasma osteocalcin and CTX were measured using an enzyme radiometric assay (Biomedical Technologies, Soughton, MA) and an enzyme linked immunosorbant assay (Immunodiagnostic Systems Inc., Fountain Hills, AZ), respectively [29].

### Osteocyte Isolation

Homozygous DMP1-GFP transgenic mice (provided by D. W. Rowe) [49] were crossed with hemizygous DMP1-caPTHR1 mice to generate DMP1-GFP-positive offspring with and without the DMP1-caPTHR1 transgene. Calvaria cells were isolated from 3- to 6-day-old mice using a modification of a previously described protocol [15], [29], [79]. Briefly, bones from each mouse were individually subjected to serial trypsin/collagenase digestions of 20 minutes each and fractions 2 through 9 were collected. Cells obtained from mice exhibiting the same genotype were pooled and GFP-expressing cells (enriched in osteocytes) were separated from GFP-negative cells (enriched in osteoblasts) by immediately subjecting the cell suspension to sorting using a FACSAria flow cytometer (BD Biosciences, San Jose, CA) at the University of Arkansas for Medical Sciences Flow Cytometry Core Facility. RNA was isolated from the cells immediately after sorting.

### Gene Expression Studies

#### Quantitative PCR

Total RNA was purified from cells or tissues using Ultraspec reagent (Biotecx Laboratories, Houston, TX), according to the manufacturer's instructions. Taqman quantitative RT-PCR was performed as previously described [75] using primer probe sets from Applied Biosystems (Foster City, CA). Relative mRNA expression levels were normalized to the house-keeping gene ribosomal protein S2 using the ΔCt method [80].

#### Western Blot Analysis

Immunoblots were performed on protein lysates prepared from vertebral bone (L6) as previously described [15]. One hundred µg of protein lysate from individual mice was separated on a 10% SDS gel and then transferred to Immobilon membrane (Millipore, Billerica, MA). The membrane was then incubated with goat polyclonal anti-mouse sclerostin antibody (1∶100 in 5% non fat milk) overnight at 4°C, followed by incubation with rabbit anti-goat-HRP secondary antibody (1∶2000 in 5% milk) for 1 hour at room temperature, and then developed with enhanced chemiluminescence (Pierce Biotechnology, Inc., Rockford, IL).

#### Immunohistochemistry

Ulnae were fixed in neutral buffered formalin, decalcified, and then embedded in paraffin. Eight µm sections were incubated with goat polyclonal anti-mouse sclerostin antibody (R&D Systems, Minneapolis, MN) diluted 1∶100 in 2% rabbit serum, followed by rabbit anti-goat-HRP conjugated secondary antibody (Santa Cruz Biotechnologies, Santa Cruz, CA) diluted 1∶200 in 2% rabbit serum, and developed with a DAB substrate-chromogen system (Dako Corp., Carpinteria, CA), as previously described [15], [73]. Sections were washed and counterstained with methyl green.

#### TCF-βGAL Reporter Gene Assay

Homozygous TCF-βGAL transgenic mice (Jackson Laboratories, Bar Harbor, ME) [81], which contain a β-galactosidase reporter gene under the control of a TCF-responsive promoter, were crossed with hemizygous DMP1-caPTHR1 mice to obtain TCF-βGAL mice with or without the DMP1-caPTHR1 transgene. β-galactosidase activity was determined in femurs from 31-day-old mice using the Galacto-Light kit (Applied Biosystems, Foster City, CA), as previously described [82]. Bones were homogenized for 20 seconds in lysis buffer and protein content was determined using the BioRad DC protein assay kit (Hercules, CA). β-galactosidase activity was expressed as relative fluorescent units/200 µg protein.

#### Assay for osteoblast progenitors

Bone marrow cells were isolated from 4.5-week-old wild type and transgenic mice (8 mice per group). Four sets of 2 samples from different mice were pooled and seeded at a density of 0.3×10^6^ cells/cm^2^. CFU-F and CFU-OB were developed in the presence of 1 mM L-ascorbic acid phosphate magnesium salt n-hydrate (A2P, Wako Chemicals USA, Richmond, VA) for 10 and 25 days, respectively, as previously published [83]. For the determination of CFU-F, colonies were stained for alkaline phosphatase and counterstained with hematoxylin. Colonies of cells containing a minimum of 20 cells were designated as CFU-F. For the determination of CFU-OB, cultures were stained using Von Kossa staining to visualize and enumerate colonies containing mineralized bone matrix.

### Statistical Analysis

Data were analyzed using SigmaStat (SPSS Science, Chicago, IL). All values are reported as the mean±SD. Differences between group means were evaluated with Student's t test or ANOVA.

## Supporting Information

Figure S1 Generation of DMP1-caPTHR1 Transgenic Mice (A) Expression of the human PTHR1 (transgene), murine PTHR1, and Sost, normalized to ribosomal protein S2, was determined by quantitative RT-PCR in osteoblast-enriched (GFP−) and osteocyte-enriched (GFP+) cell preparations from wild type or DMP1-caPTHR1 transgenic mice. (B) Total body weight of female and male DMP1-caPTHR1 transgenic mice and wild type littermates measured every 4 weeks up to 24 weeks of age. Symbols represent the mean±S.D. of 5 mice. (C) Serial measurement of femoral and spinal BMD of male DMP1-caPTHR1 mice and wild type littermates. Symbols represent the mean±S.D. of 3–5 mice. * p<0.05 vs. WT mice for each time point. (D) Micro-CT image of femurs from 8-month-old wild type and DMP1-caPTHR1 mice. (E) Total, femoral, and spinal BMD in 9-week-old WT and DMP1-caPTHR1 male mice from an independent transgenic line, 559. Bars represent the mean±S.D. of 5 mice. * p<0.05 vs. WT mice.
(0.19 MB TIF)

Figure S2 Micro-CT Analysis of 3-Day-Old and 3.5-Week-Old DMP1-caPTHR1 Transgenic Mice (A) Representative longitudinal and cross-sectional micro-CT images of femurs from 3-day-old DMP1-caPTHR1 mice (TG) and wild type littermates (WT). Three animals per genotype were analyzed. The total bone volume per femur was measured. * indicates p<0.05 vs wild type littermates. (B) Longitudinal and cross-sectional micro-CT images of femurs, 5th lumbar vertebra, and calvaria from 3.5-week-old WT and TG mice.
(0.35 MB TIF)

Figure S3 Hematoxylin & Eosin Staining of Longitudinal Sections of Femoral Diaphysis of 4-, 8-, and 12-Week-Old DMP1-caPTHR1 Transgenic Mice Decalcified, paraffin embedded femurs were longitudinally sectioned and stained with hematoxylin and eosin. Bars indicate 0.1 mm.
(2.00 MB TIF)

Figure S4 Circulating levels of Calcium, Phosphate and PTH are not altered in DMP1-caPTHR1 transgenic mice Plasma levels of (A) total calcium, (B) inorganic phosphate, and (C) PTH in DMP1-caPTHR1 mice and wild type littermates. Bars are mean±S.D. (A) n = 4 wild type and 5 transgenic 3.5-week-old; (B) n = 11 wild type and 16 transgenic 2.5-month-old mice; and (C) n = 5 wild type and 7 transgenic 2-month-old mice.
(0.09 MB TIF)

Figure S5 Osteoblast and Osteoclast Markers are Increased in DMP1-caPTHR1 Transgenic Mice (A) Quantitative RT-PCR analysis of osteoblastic genes in tibia from 9-week-old DMP1-caPTHR1 transgenic mice and wild type littermates. Bars represent the mean±SD of 3 mice. *p<0.05 vs. WT mice. (B) In situ hybridization of tibia sections from 10.5-week-old mice with the indicated probes. (C) Quantitative RT-PCR of osteoclast-specific genes in tibia of 9-week-old mice. Bars represent the mean±SD of 3 mice. * p<0.05 vs. WT mice.
(1.03 MB TIF)

Text S1 (0.03 MB DOC)

Table S1 (0.04 MB DOC)
