# Supplementary figures and images for: Control of Bone Mass and Remodeling by PTH Receptor Signaling in Osteocytes
Source: PLoS One. 2008 Aug 13;3(8):e2942. doi: 10.1371/journal.pone.0002942 (PMC2491588; doi:10.1371/journal.pone.0002942)

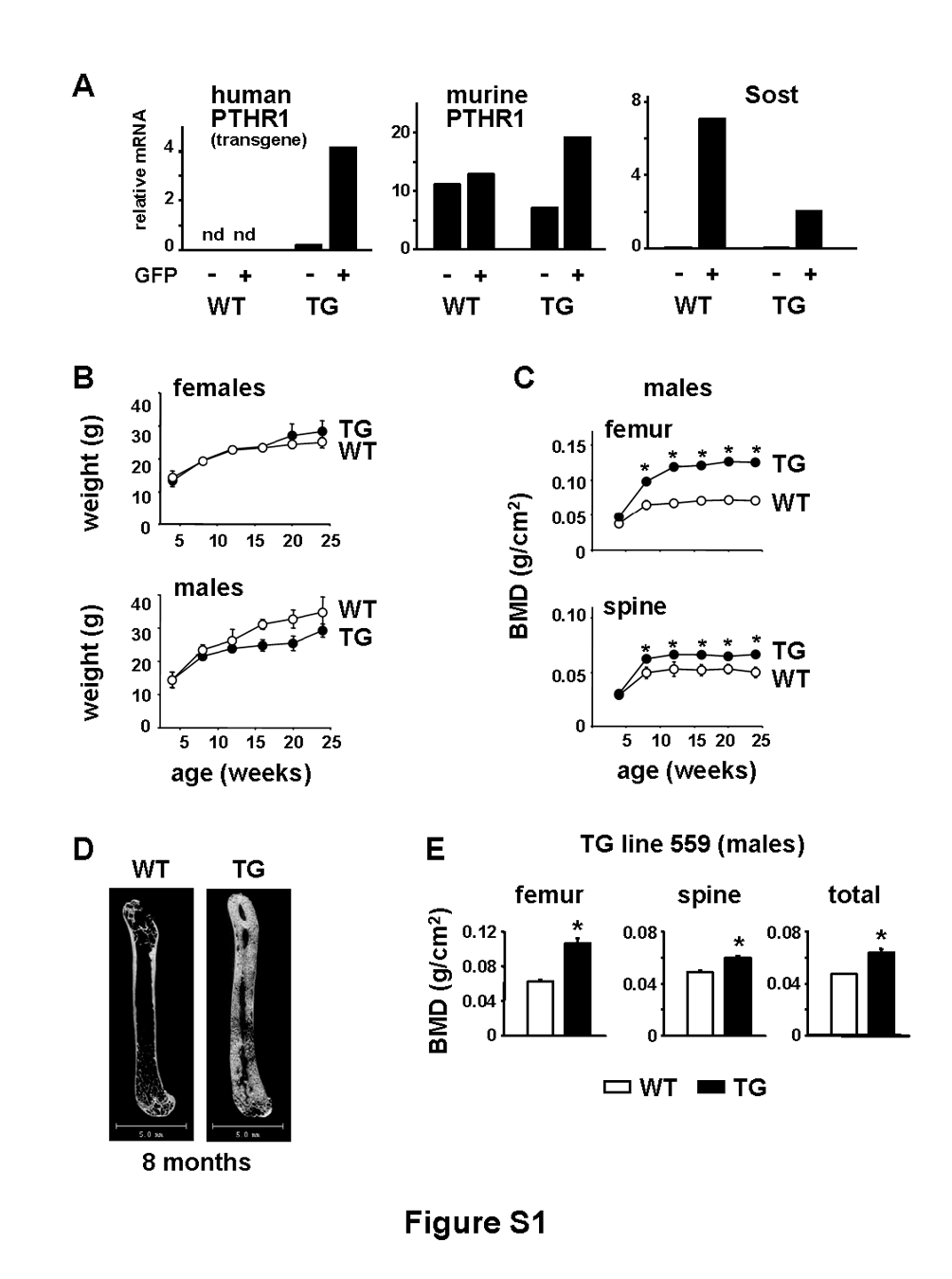

Supplement: Figure S1 — Generation of DMP1-caPTHR1 Transgenic Mice (A) Expression of the human PTHR1 (transgene), murine PTHR1, and Sost, normalized to ribosomal protein S2, was determined by quantitative RT-PCR in osteoblast-enriched (GFP−) and osteocyte-enriched (GFP+) cell preparations from wild type or DMP1-caPTHR1 transgenic mice. (B) Total body weight of female and male DMP1-caPTHR1 transgenic mice and wild type littermates measured every 4 weeks up to 24 weeks of age. Symbols represent the mean±S.D. of 5 mice. (C) Serial measurement of femoral and spinal BMD of male DMP1-caPTHR1 mice and wild type littermates. Symbols represent the mean±S.D. of 3–5 mice. * p<0.05 vs. WT mice for each time point. (D) Micro-CT image of femurs from 8-month-old wild type and DMP1-caPTHR1 mice. (E) Total, femoral, and spinal BMD in 9-week-old WT and DMP1-caPTHR1 male mice from an independent transgenic line, 559. Bars represent the mean±S.D. of 5 mice. * p<0.05 vs. WT mice. (0.19 MB TIF) [file pone.0002942.s001.tif]

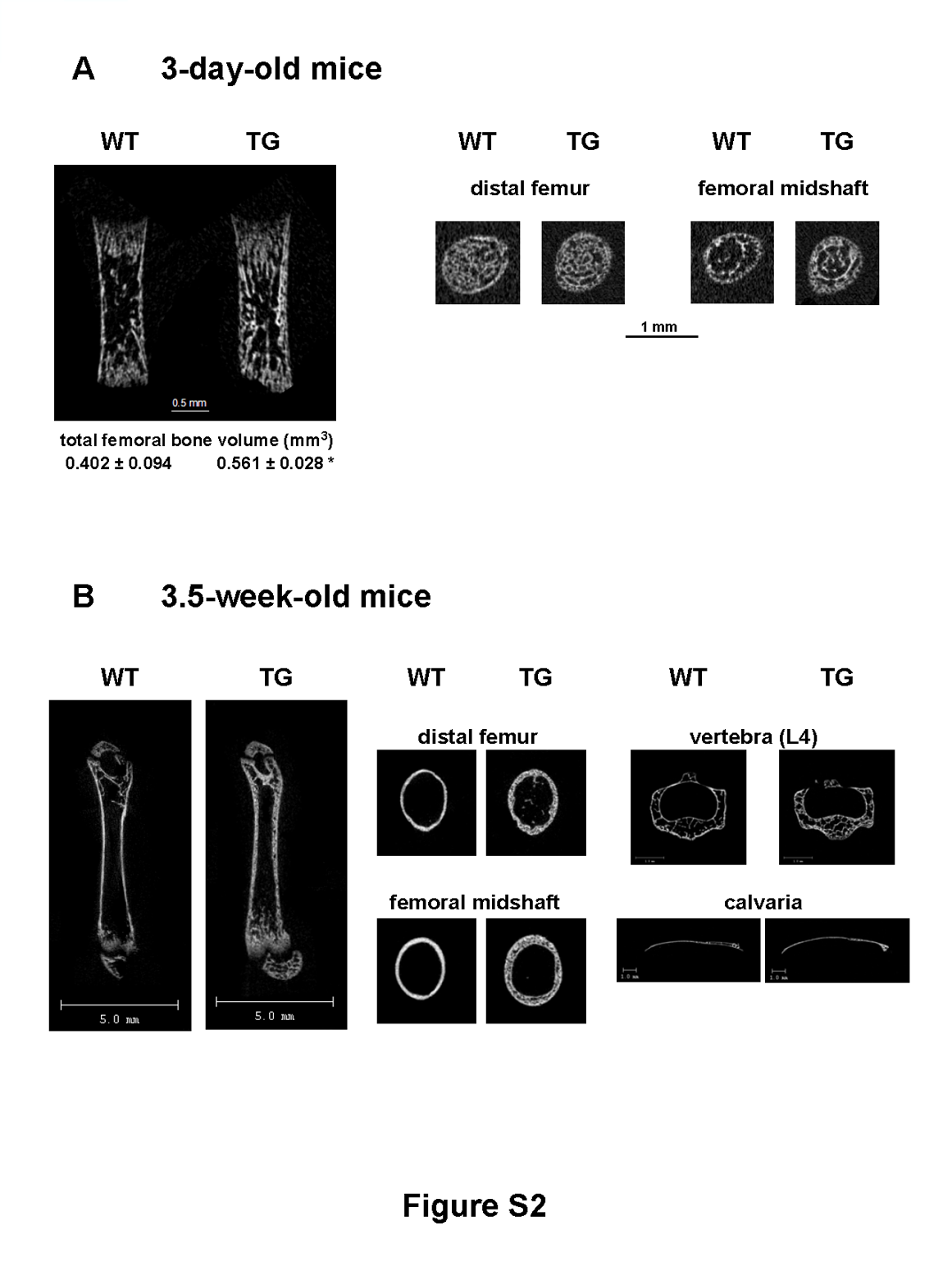

Supplement: Figure S2 — Micro-CT Analysis of 3-Day-Old and 3.5-Week-Old DMP1-caPTHR1 Transgenic Mice (A) Representative longitudinal and cross-sectional micro-CT images of femurs from 3-day-old DMP1-caPTHR1 mice (TG) and wild type littermates (WT). Three animals per genotype were analyzed. The total bone volume per femur was measured. * indicates p<0.05 vs wild type littermates. (B) Longitudinal and cross-sectional micro-CT images of femurs, 5th lumbar vertebra, and calvaria from 3.5-week-old WT and TG mice. (0.35 MB TIF) [file pone.0002942.s002.tif]

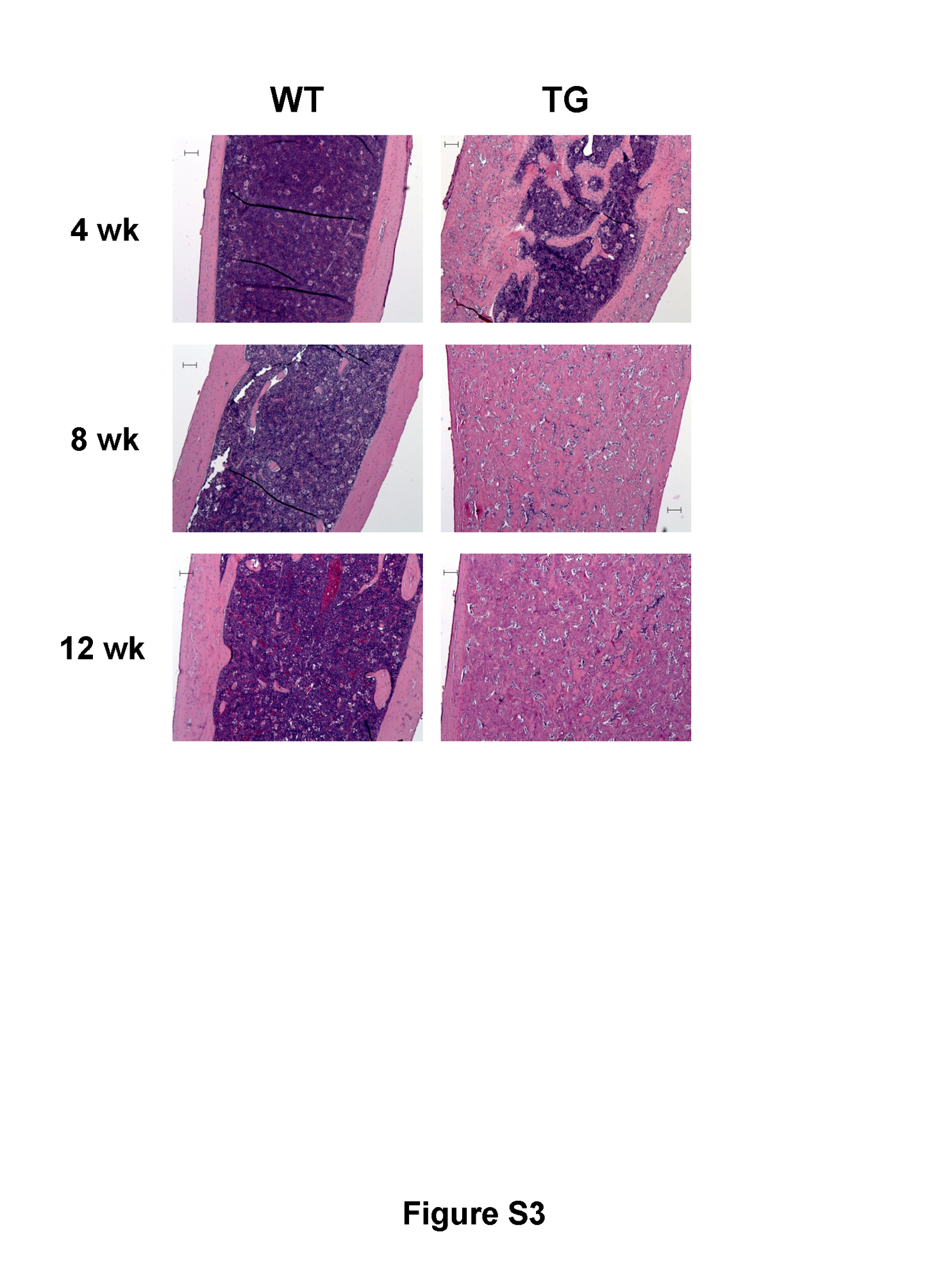

Supplement: Figure S3 — Hematoxylin & Eosin Staining of Longitudinal Sections of Femoral Diaphysis of 4-, 8-, and 12-Week-Old DMP1-caPTHR1 Transgenic Mice Decalcified, paraffin embedded femurs were longitudinally sectioned and stained with hematoxylin and eosin. Bars indicate 0.1 mm. (2.00 MB TIF) [file pone.0002942.s003.tif]

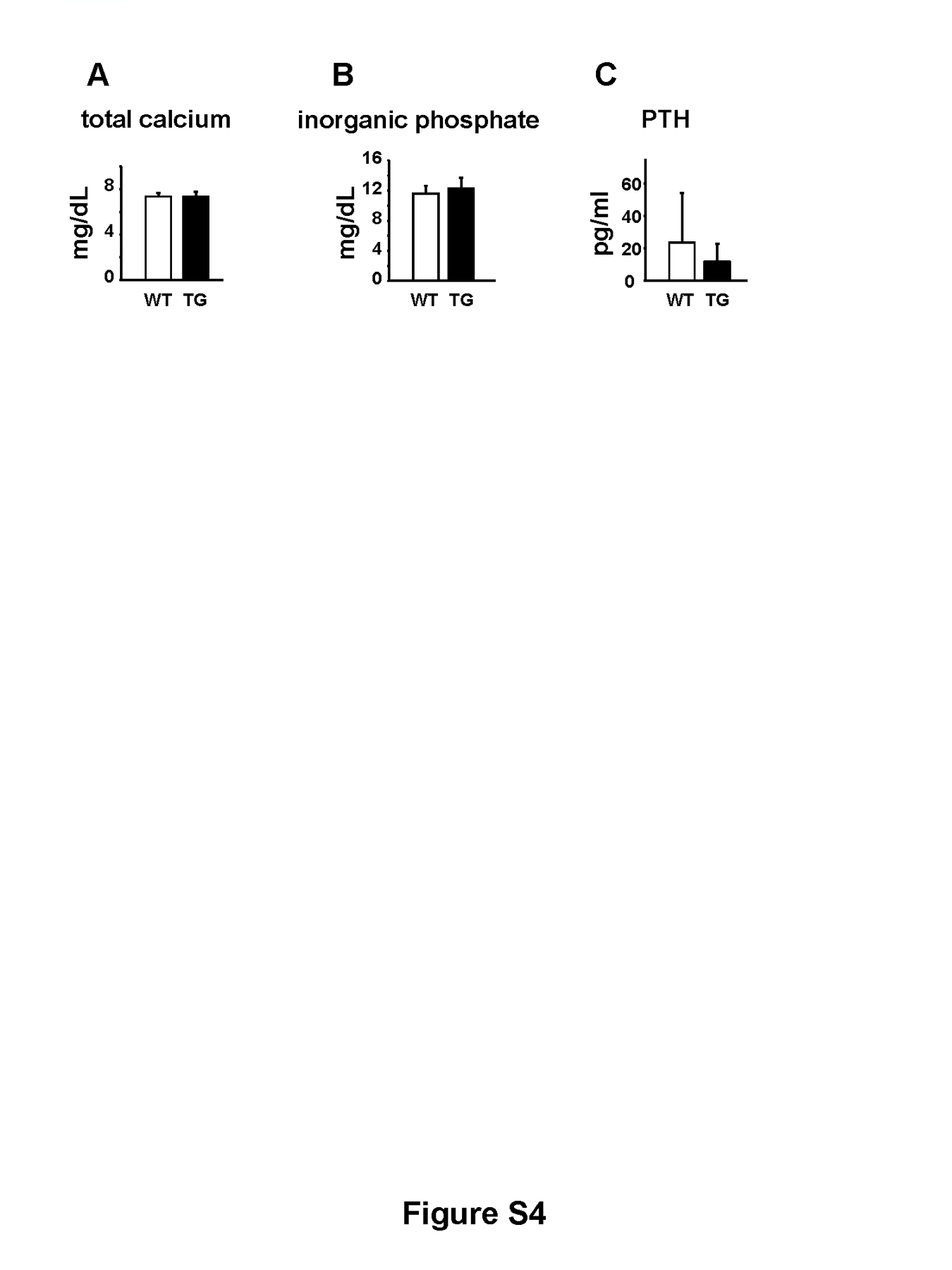

Supplement: Figure S4 — Circulating levels of Calcium, Phosphate and PTH are not altered in DMP1-caPTHR1 transgenic mice Plasma levels of (A) total calcium, (B) inorganic phosphate, and (C) PTH in DMP1-caPTHR1 mice and wild type littermates. Bars are mean±S.D. (A) n = 4 wild type and 5 transgenic 3.5-week-old; (B) n = 11 wild type and 16 transgenic 2.5-month-old mice; and (C) n = 5 wild type and 7 transgenic 2-month-old mice. (0.09 MB TIF) [file pone.0002942.s004.tif]

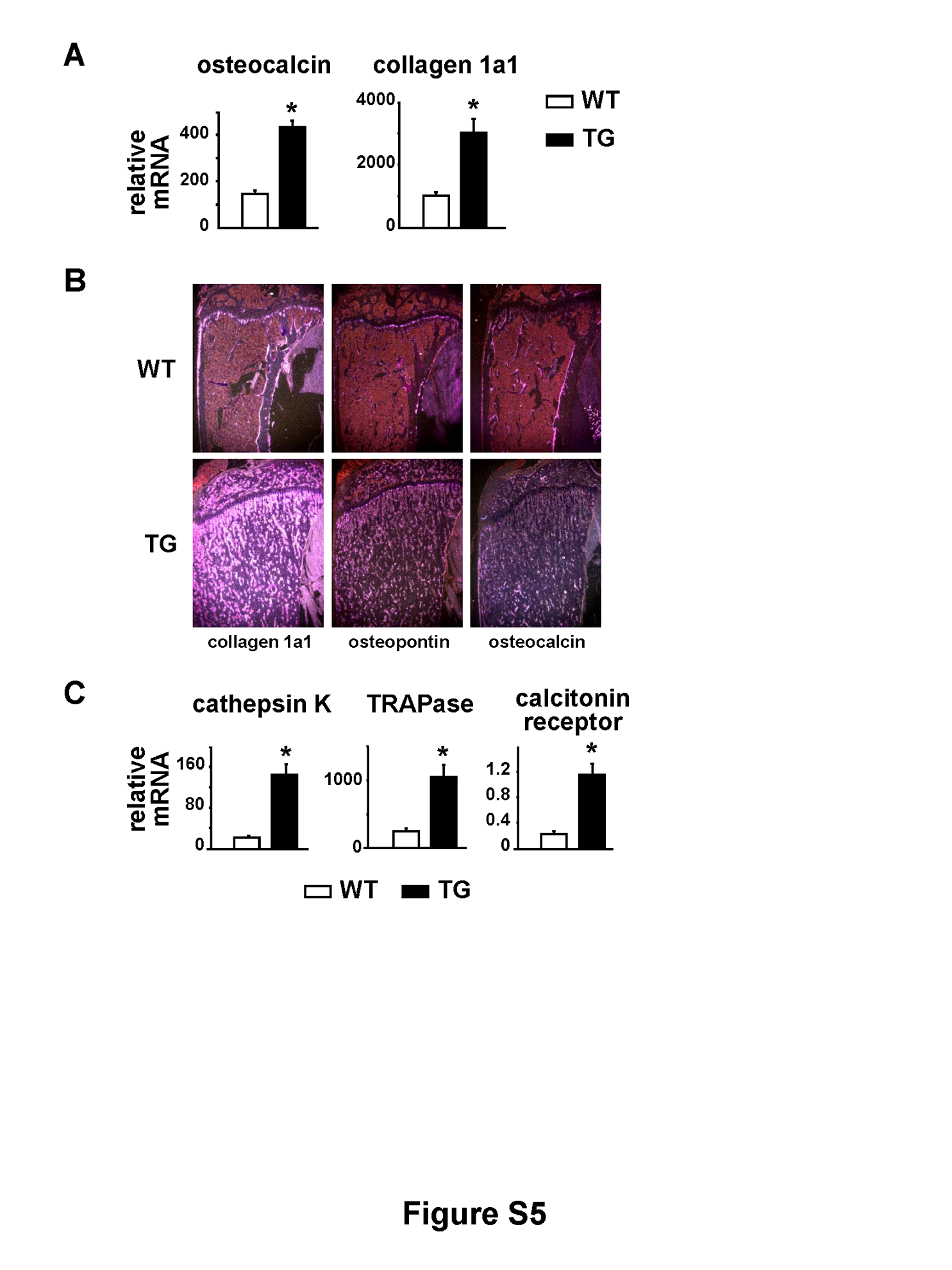

Supplement: Figure S5 — Osteoblast and Osteoclast Markers are Increased in DMP1-caPTHR1 Transgenic Mice (A) Quantitative RT-PCR analysis of osteoblastic genes in tibia from 9-week-old DMP1-caPTHR1 transgenic mice and wild type littermates. Bars represent the mean±SD of 3 mice. *p<0.05 vs. WT mice. (B) In situ hybridization of tibia sections from 10.5-week-old mice with the indicated probes. (C) Quantitative RT-PCR of osteoclast-specific genes in tibia of 9-week-old mice. Bars represent the mean±SD of 3 mice. * p<0.05 vs. WT mice. (1.03 MB TIF) [file pone.0002942.s005.tif]
