# Supplementary material for: Control of Bone Mass and Remodeling by PTH Receptor Signaling in Osteocytes
Source: PLoS One. 2008 Aug 13;3(8):e2942. doi: 10.1371/journal.pone.0002942 (PMC2491588; doi:10.1371/journal.pone.0002942)
Supplement: Text S1 — (0.03 MB DOC) [file pone.0002942.s006.doc]

**SUPPLEMENTARY TEXT**

**METHODS**

***In Situ* Hybridization.** *In situ* hybridizations were performed as described [39] using ^35^S-labeled riboprobes transcribed from plasmids encoding human pro-alpha 1(I) chain of type I procollagen, mouse osteopontin, and mouse osteocalcin. Sections were counterstained with hematoxilin and eosin.

**Calcium, Phosphate and PTH measurements**

Plasma total calcium and phosphate were measured by colorimetric determination (Stanbio Lab, Boerne, TX and BioAssay Systems, Hayward, CA, respectively). Plasma PTH levels were measured using an intact rodent PTH immunoassay (Immutopics, San Clemente, CA). Urine DPD and creatinin were measured using a competitive enzyme immunoassay and a colorimetric assay, respectively (Quidel, San Diego, CA).
