# Supplementary material for: Control of Bone Mass and Remodeling by PTH Receptor Signaling in Osteocytes
Source: PLoS One. 2008 Aug 13;3(8):e2942. doi: 10.1371/journal.pone.0002942 (PMC2491588; doi:10.1371/journal.pone.0002942)
Supplement: Table S1 — (0.04 MB DOC) [file pone.0002942.s007.doc]

**Table S1. Histomorphometric Analysis of Femoral Cancellous Bone of 3.5-Week-Old DMP1-caPTHR1 Mice**

|  | units | WT | TG |
| --- | --- | --- | --- |
| bone area/tissue area | % | 9.76 ± 3.62 | 21.20 ± 4.92* |
| trabecular width | μm | 22.74 ± 0.89 | 39.31 ± 7.44* |
| trabecular separation | μm | 238.92 ± 120.66 | 148.51 ± 29.68 |
| trabecular number | /mm | 4.32 ± 1.64 | 5.40 ± 0.79 |
| osteoid area/bone area | % | 2.22 ± 0.53 | 4.09 ± 2.59 |
| osteoid perimeter/bone perimeter | % | 6.18 ± 1.53 | 16.35 ± 1.39* |
| osteoid width | μm | 4.29 ± 0.52 | 6.85 ± 0.71* |
| osteoblast perimeter/bone perimeter | % | 14.62 ± 2.45 | 32.44 ± 3.13* |
| osteoblast number/bone perimeter | /mm | 13.90 ± 4.95 | 33.28 ± 4.12* |
| osteoblast number/bone area | /mm^2^ | 121.41 ± 75.09 | 358.29 ± 60.71* |
| osteoclast perimeter/bone perimeter | % | 2.34 ± 0.55 | 5.19 ± 1.34* |
| osteoclast number/bone perimeter | /mm | 0.84 ± 0.27 | 1.71 ± 0.52* |
| osteoclast number/bone area | /mm^2^ | 7.82 ± 5.16 | 18.14 ± 3.89* |
| osteocyte number/bone area | /mm^2^ | 5.58 ± 1.37 | 6.29 ± 1.63 |
| quiescence perimeter/bone perimeter | % | 89.61 ± 2.81 | 74.26 ± 2.10* |

* p ≤ 0.05 vs. WT by *t-test*, n=3
